# Dietary Supplementation of a Live Yeast Product on Dairy Sheep Milk Performance, Oxidative and Immune Status in Peripartum Period

**DOI:** 10.3390/jof6040334

**Published:** 2020-12-03

**Authors:** Alexandros Mavrommatis, Christina Mitsiopoulou, Christos Christodoulou, Dimitris Karabinas, Valentin Nenov, George Zervas, Eleni Tsiplakou

**Affiliations:** 1 Laboratory of Nutritional Physiology and Feeding, Department of Animal Science, School of Animal Biosciences, Agricultural University of Athens, Iera Odos 75, GR-11855 Athens, Greece; mavrommatis@aua.gr (A.M.); chr_mitsiopoulou28@hotmail.com (C.M.); c.christodoulou@aua.gr (C.C.); vdkarabinas@vkarabinas-sa.gr (D.K.); gzervas@aua.gr (G.Z.); 2 Phileo Lesaffre Animal Care, Marcq en Baroeul, 59700 Nord, France; info@phileo.lesaffre.com

**Keywords:** *Saccharomyces cerevisiae*, livestock, ewes, energy, antioxidant, cytokines

## Abstract

This study evaluated the dietary administration of *Saccharomyces cerevisiae* live yeast on milk performance and composition, oxidative status of both blood plasma and milk, and gene expression related to the immune system of lactating ewes during the peripartum period. Chios ewes were fed either a basal diet (BD) (Control, *n* = 51) or the BD supplemented with 2 g of a live yeast product/animal (ActiSaf, *n* = 53) from 6 weeks prepartum to 6 weeks postpartum. Fatty acid profile, oxidative, and immune status were assessed in eight ewes per treatment at 3 and 6 weeks postpartum. The β-hydroxybutyric acid concentration in blood of ActiSaf fed ewes was significantly lower in both pre- and postpartum periods. A numerical increase was found for the milk yield, fat 6% corrected milk (Fat corrected milk (FCM_6%_)), and energy corrected milk yield (ECM) in ActiSaf fed ewes, while daily milk fat production tended to increase. The proportions of C_15:0_, C_16:1_, C_18:2n6t_, and C_18:3n3_ fatty acids were increased in milk of ActiSaf fed ewes, while C_18:0_ was decreased. Glutathione reductase in blood plasma was increased (*p* = 0.004) in ActiSaf fed ewes, while total antioxidant capacity measured by 2,2′-Azino-bis (3-ethylbenzthiazoline-6-sulfonic acid) (ABTS) method was decreased (*p* < 0.001). Higher ABTS values were found in the milk of the treated group. The relative transcript levels of *CCL5*, *CXCL16,* and *IL8* were suppressed, while that of *IL1B* tended to decrease (*p* = 0.087) in monocytes of ActiSaf fed ewes. In conclusion, the dietary supplementation of ewes with *S. cerevisiae*, improved the energy utilization and tended to enhance milk performance with simultaneous suppression on mRNA levels of pro-inflammatory genes during the peripartum period.

## 1. Introduction

Both meat and dairy products consumption are expected to increase in 2050 by 73 and 58%, respectively, compared to their 2010 levels [1,2], due to the rapid population growth rate. Ruminants’ milk (67%) and meat (33%) cover 51% of proteins derived from the livestock sector and have a dominant role in food security, which is linked to how efficient animals utilize feed. Ruminants’ feed efficiency depends upon the microbes residing within the rumen that ferment and transform feeds into volatile fatty acids (VFAs), proteins, and vitamins which are exploited by the host [3]. This multikingdom ecosystem’s efficiency is dependent on various factors, the most prominent being that of diet. The improvement of the rumen microbiome habitat through the advancement of feed efficiency technologies entails a fundamental stepping stone in the overall improvement of livestock systems sustainability and food security concerns.

In intensive farming systems, high genetic merit animals require higher amounts of concentrate to fulfil their energy and nutrient demands, resulting in metabolic imbalances in rumen function and their microbiome governance. Probiotic yeasts are currently popular and widely used in ruminant feeding systems, especially since some of them have been officially authorized as feed additives in Europe [4]. The main purpose for using such additives in ruminant diets is to prevent rumen flora disorders and disturbances [5]. Dietary supplementation with live yeast (LY), *Saccharomyces cerevisiae*, improves rumen function through several modes of action [6]. This improvement is related to the oxygen scavenging properties of yeast in rumen (anaerobiosis mechanism), which upgrades bacterial viability and therefore the animal production [6]. Amongst the favorable bacteria are cellulolytics, which through the increase in their activity enhance fiber digestion. Moreover, LY can also stabilize the ruminal pH [7], not only after feeding, but also during the peripartum period where animals often find themselves in a negative energy balance and are further sensitive to metabolic diseases. It has been proven that even a low-grade energy deficiency weakens the animals’ antioxidant system, which fails to neutralize the formation of Reactive Oxygen Species (ROS) and triggers the pro-inflammatory response [8,9].

By improving ruminants’ feed utilization and both energy and nutrient availability during the peripartum period, not only can milk performance and chemical composition be enhanced, but furthermore a downregulation in the immunostimulation response can be achieved through the limitation of lipomobilization metabolites [10,11]. Although LY supplementation in ruminants’ diets is a well-established nutritional strategy, previous works have only focused on district parameters instead of a holistic approach. Specifically, except for milk performance [7,10,11,12,13,14], scarce information has been linked to the potential improvement of energy balance and oxidative status and therefore to the immune response under the influence of dietary yeasts inclusion in ruminants.

Taking into account the aforementioned information, the objective of this work was to evaluate the effect of LY *S. cerevisiae* (CNCM I-4407, 10^10^ CFU/g, ActiSaf; Phileo Lesaffre Animal Care, France) in dairy sheep during the transition and early lactation period (6 weeks prepartum and 6 weeks postpartum) on milk performance and composition, antioxidant status (determined by Glutathione transferase (GST), Glutathione reductase (GR), Superoxide dismutase (SOD), Glutathione peroxidase (GSH-Px), Catalase (CAT) and Lactoperoxidase (LPO) activities, antioxidant capacity with 2,2′-Azino-bis (3-ethylbenzthiazoline-6-sulfonic acid) (ABTS) and Ferric reducing ability of plasma (FRAP) methods and oxidative stress indicators such as Malondialdehyde (MDA) and protein carbonyls (PCs)) on both milk and blood plasma and key-gene expression (*CCL5, CXCL16, INFG, IL1B, IL2, IL6, IL8, IL10, TNF, NFKB*) in monocytes and neutrophils which are associated with cytokine production.

## 2. Materials and Methods

### 2.1. Location and Environmental Conditions

The experiment was conducted from November 2019 to March 2020 on a commercial dairy sheep farm in the region of Chiliomodi in Korithia, Greece. This region has a typical Mediterranean climate with hot dry summers and relatively mild wet winters. During the experimental period, the mean temperatures in November, December, January, February, and March were 12.2, 8, 9.3, 11.1 and 13.1 °C, respectively. The selected farm represents the typical intensive dairy sheep production system of Greece.

### 2.2. Animals and Diets

Animals’ housing, management, handling, and care complied with the latest European Union Directive on the protection of animals used for scientific purposes [15], while taking into account an extended experimental design report, the Bioethical Committee of Faculty of Animal Science (currently known as the Agricultural University of Athens Ethical Committee in Research; FEK 38/Α/2-3-2018, eide AUA) approved the experimental protocol. One hundred and twenty (120), 1- to 3-year-old dairy ewes (*Ovis aries*), of pure Chios breed, were physically selected from a flock of six hundred. At approximately 6 weeks before parturition, the ewes were divided into two homogenous groups based on their body weight (BW), number of parturition, and the milk yield from the previous year only for the case of multiparous ewes (2.1 ± 0.68 kg). Both groups had the same number of primi- (*n* = 20) and multiparous (*n* = 40) animals. More specifically, the ewes mean BW in the Control group (*n* = 60) was 61.5 ± 10.70 (SD) kg while in the ActiSaf group (*n* = 60) was 61.5 ± 11.02 (SD) kg. The Control group was fed a basal diet comprising of concentrate mix, alfalfa hay, and oat hay, while the ActiSaf group consumed the same basal diet supplemented with 2 g of *S. cerevisiae* LY/day/ewe (CNCM I-4407, 10^10^ CFU/g, ActiSaf; Phileo Lesaffre Animal Care, France) (Table 1). The animals were housed in two pens based on the dietary treatment. Both diets were isonitrogenous and isocaloric and were designed to meet ewes’ requirements in the transition period and early lactation according to the flock fat (6%) corrected milk yield [16,17]. The animals were fed on a group basis while forages were offered separately from the concentrate in three equal portions after milking. Diet selectivity did not occur, no refusals of forages and/or concentrate were observed, and all animals had free access to fresh water. The experimental procedure lasted 6 weeks started from each ewes’ parturition. After this, each ewe was returned to the commercial farm flock and the experiment ended when the final ewe had completed its 6th week on lactation. Since milk performance was recorded at the same time points, lactation stage had no effect on milk performance. Control ewes (*n* = 60) gave birth to 141 lambs (prolificacy = 2.35; 69 females and 72 males) while those of the ActiSaf (*n* = 60) gave birth to 142 (prolificacy = 2.36; 65 females and 77 males). In addition, since the experimental trial took place on farm-scale conditions, few ewes were unable to be exploited for data curation due to abortions (4), mastitis (10) or dystocia (2), hence the final number of subjects was re-adjusted to 51 and 53 for the Control and ActiSaf groups, respectively.

### 2.3. Feed Samples Analyses

Samples of the alfalfa hay, oat hay, and concentrate were analyzed for organic matter (OM; Official Method 7.009), dry matter (DM; Official Method 7.007), and crude protein (CP; Official Method 7.016) according to the Association of Official Analytical Chemists (1984) using a Kjeldahl Distillation System (FOSS Kjeltec 8400, Demark). Neutral detergent fiber (NDF) and acid detergent fiber (ADF) expressed exclusive of residual ash according to the method of Van Soest using an ANKOM 2000 Fiber Analyzer (USA) as described by Tsiplakou et al. [18] (Table 1).

### 2.4. Milk Samples Collection

The sheep were milked three times per day (at 0700, 1300 and 2000 h) with a milking machine equipped with a digital milk meter and an electronic identification system (Sylco, Greece); thus, milk yield was recorded daily, and software (Sylco, Greece) was set to provide weekly averages. Milk samples were collected from each ewe weekly (at 7, 14, 21, 28, 35 and 42 days from parturition) for a 6-week period with sampling bottles (Sylco, Greece) of 200 mL appropriately for the milking parlor, to receive a representative sample of the milked quantity. Each of the milk samples from the mix of three subsamples was derived from each milking time (at 0700, 1300 and 2000 h) by taking 5% of the milked quantity.

### 2.5. Milk Chemical Composition

The milk samples were analyzed for fat, protein, lactose, total solids, and total solids no-fat by IR spectrometry (MilkoScan 120; FOSS, Hillerød, Demark) after proper calibration according to the methods of Gerber [19] and Kjeldahl [20].

### 2.6. Blood Metabolic Biomarker (B-HBA) Determination

Four weeks before the expected parturition, blood B-HBA was individually determined (before the morning feeding, 0700 h) once every three days until the lambing to ensure that 15 days before parturition a measurement would be recorded (Table S1). Two weeks postpartum, the sample collection for B-HBA was repeated. Blood ketone concentrations were measured using an electrochemical capillary blood monitoring device (FreeStyle Precision Neo, Abbott Laboratories Hellas S.A) with the corresponding individual foil-wrapped test strips for B-HBA. This method of B-HBA determination possesses 98.4% accuracy for the prediction of both toxemias’ pregnancy and ketosis in Chios ewes [21]. After the insertion of a test strip into the device, a drop of blood was applied to the assigned spot, and the B-HBA concentration was recorded. Data were interpreted using 208 determinations in the two aforementioned sampling time points.

### 2.7. Antioxidant Status, Immune Response, and Milk Fatty Acid Profile

Eight (*n* = 8) ewes of each group with comparable weights (Control: 60.2 ± 5.11 kg; ActiSaf: 60.3 ± 4.88 kg), ages (Control: 1.84 ± 0.16 kg; ActiSaf: 1.85 ± 0.18 kg), milk performance (Fat corrected milk 6% (FCM_6%_) data used up 14th day in milk (DIM), Control: 2.3 ± 0.15 kg; ActiSaf: 2.3 ± 0.21 kg), prolificacy (Control: 2; ActiSaf: 2), and same lactation stage (up to 3 days deviation between animals) were selected for determining the antioxidant status of both milk and blood, for immune system gene response, and for milk fatty acid profile. Milk samples were collected (as mentioned above) in the 3rd and 6th week postpartum and stored at −80 °C. Blood samples were also collected, before the morning feeding (0700 h), at the same time points in heparin contained tubes for cell extraction and plasma isolation.

### 2.8. Enzyme Assays, Oxidative Stress Biomarkers, and Total Antioxidant Capacity

The enzyme activities, oxidative stress biomarkers, and the total antioxidant capacity were measured spectrophotometrically (Helios alpha, UNICAM, Cambridge, UK) as previously described by Tsiplakou et al. [22]. Briefly, Glutathione transferase (GST) activity in blood plasma was measured according to the method described by Labrou et al. [23] by measuring the conjunction of reduced glutathione to 1-chloro-2,4-dinitrobenzene at 340 nm. Catalase (CAT) activity in blood plasma and milk were assessed using a continuous spectrophotometric rate for the determination of H_2_O_2_ at 520 nm, according to the Sigma-Aldrich Catalase Assay Kit (CAT100). Glutathione peroxidase (GSH-Px) activity in blood plasma was measured according to the method of Paglia and Valentine [24] at 340 nm. Glutathione reductase (GR) activities in both blood plasma and milk were measured according to the method of Mavis and Stellwagen [25] by measuring the reduction in oxidized glutathione at 340 nm. Superoxide dismutase (SOD) activities in both blood plasma and milk were assayed using the method of McCord and Fridovich [26] by measuring the inhibition of cytochrome c oxidation at 550 nm. Lactoperoxidase (LPO) activity in milk was performed according to the methods of Keesey [27] by measuring the oxidation of ABTS present in hydrogen peroxide at 340 nm. Malondialdehyde (MDA) was determined according to the method of Nielsen et al. [28] with some modifications. More specifically, 100 μL blood plasma was added to 700 μL ortho-phosphoric acid (Panreac ITW Companies) and 200 μL aquarius thiobarbituric acid (TBA, Sigma-Aldrich CO USA) and then the samples were heated at 100 °C for 60 min. In milk samples, 1 mL of raw milk was added to 7 mL ortho-phosphoric acid (Panreac ITW Companies) and 2 mL of aquarius TBA (thiobarbituric acid, Sigma-Aldrich CO USA) and then incubated at 100 °C for 60 min. After that, absorbance was recorded at 532 nm. The protein carbonyl (PC) content was determined according to the method of Patsoukis et al. [29] by measuring the conjunction of 2,4-dinitrophenylhydrazine (DNPH) on protein carbonyls at 375 nm. The 2,2′-Azino-bis (3-ethylbenzthiazoline-6-sulfonic acid) (ABTS) radical scavenging assay was based on the published methods [30,31]. Ferric reducing ability of plasma (FRAP) assay was used to measure total antioxidant potential according to the method described by Benzie and Strain [32].

### 2.9. Milk Fatty Acid Profile

Milk fatty acid profile was determined using Gas Chromatography (Agilent 6890 N GC, Agilent 7683 B autosampler injector), equipped with an HP-88 capillary column (60 m × 0.25 mm i. d. with 0.20 μm film thickness, Agilent Technologies, USA) and a flame ionization detector (FID) as previously described by Mavrommatis and Tsiplakou [33].

### 2.10. Monocytes and Neutrophils Immune Genes Expression

Blood monocytes and neutrophils were isolated and then total RNA was extracted as previously described by Tsiplakou et al. [34]. Pure RNA (500 ng) from 64 individual (monocytes (32) and neutrophils (32)) samples was reverse transcribed with the PrimeScript First Strand cDNA Synthesis Kit (Takara, Japan) according to the manufacturer’s instructions using a mix of random hexamers and oligo-dT primers. A pair of primers specific for each target gene was designed using Geneious software (Biomatters, New Zealand) according to the respective *Ovis aries* gene coding sequences (CDSs in GenBank) (Table S7). The specificity of each pair of primers was tested against genomic DNA (positive control) to confirm that a single amplicon would emerge after quantitative real-time PCR. In addition, dissociation curves were generated, and the amplification products were subjected to agarose gel electrophoresis to confirm the production of a single amplicon per reaction. The relative expression levels of the target genes were calculated as (1 + E)^−ΔCt^, where ΔCt is the difference between the geometric mean of the two housekeeping genes’ Cts and the Ct of the target gene, and the primer efficiency is the mean of each amplicon’s efficiency per primer, which was calculated by employing the linear regression method on the log (fluorescence) per cycle number (ΔRn) using the LinRegPCR software [35]. Glyceraldehyde 3-Phosphate Dehydrogenase (*GAPDH*) and Tyrosine 3-monoxygenase/tryptophan 5-monooxygenase activation protein, zeta polypeptide (*YWHAZ*) were used as housekeeping genes to normalize the cDNA template concentrations; the RT-PCR protocols are described in Tsiplakou et al. [18].

### 2.11. Statistical Analysis

Experimental data were analyzed using the SPSS.IBM statistical package (version 20.0) and results are presented as mean ± mean standard error (SEM). Dietary treatment effects were determined using a general linear model (GLM) for a repeated measures analysis of variance (ANOVA). With the dietary treatments (D = Control and ActiSaf) used as the fixed factor and the sampling time (S) as the repeated measure, while including their interactions (D*S) to evaluate differences over time, according to the model:

Yijkl = μ + Di + Sj + Ak + (D × S)ij + eijkl

where Υijk is the dependent variable, µ the overall mean, Di the effect of dietary treatment (i = 2; Control and ActiSaf), Sj the effect of sampling time (j = 6 for milk performance, 2 for B-HBA concentration, fatty acids profile, antioxidant and immune system), Ak the animal’s random effect, (DxS)ij the interaction between dietary treatments and sampling time, and eijk the residual error. Posthoc analysis was performed when appropriate using a Tukey’s multiple range test [36]. For all tests, the significance level was set at *p* = 0.05. In order to simplify the visualization of the results, GraphPad Prism 6.0 (2012) was used for interleaved bars while error bars represent the mean standard error (SEM).

Moreover, discriminant analysis was also applied to pooled data to establish those variables capable of distinguishing and classifying samples among the two dietary treatments. Wilk’s lambda (λ) criterion was used for selecting discriminant variables [37]. Forty variables were entered to develop a model to discriminate the thirty-two samples of each case. Specifically, five variables were used for grouped fatty acids in milk, ten and 10 for immune system genes’ relative expression in monocytes and neutrophils, respectively, and seven and eight in antioxidant indices in milk and blood, respectively.

## 3. Results

### 3.1. Animal Performance

**Figure 1:**
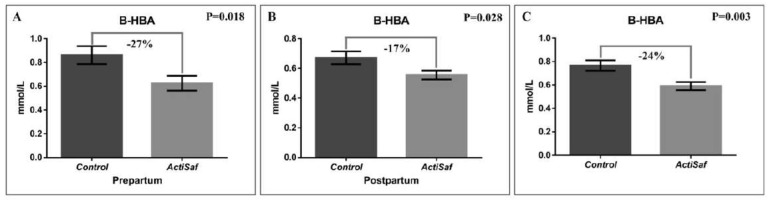
Graphical representation of (**A**) β-hydroxybutyric acid (B-HBA) in ewes’ blood prepartum (mean ± SE), (**B**) β-hydroxybutyric acid in blood postpartum (mean ± SE), and (**C**) β-hydroxybutyric acid in blood of ewes in Control (black, *n* = 51 ewes) and ActiSaf (grey, *n* = 53 ewes) groups in experimental period of 12 weeks (mean ± mean standard error (SEM)).

**Figure 2:**
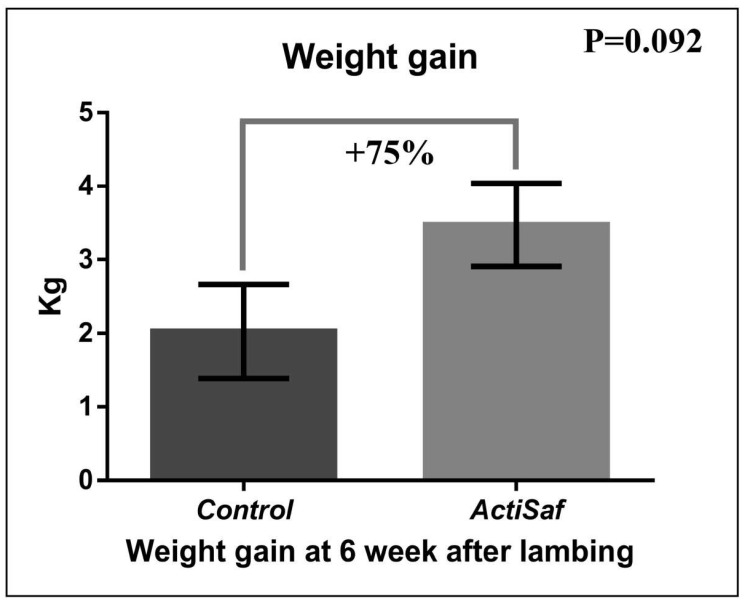
Graphical representation of body weight gain (recovery) between lambing and 6th week of lactation of ewes in Control (black, *n* = 51 ewes) and ActiSaf (grey, *n* = 53 ewes) groups (mean ± SE).

Dietary supplementation with LY ActiSaf significantly reduced the B-HBA concentrations in ewes’ blood by 27% (0.86 ± 0.07 vs. 0.63 ± 0.06 mmol/L, *p* = 0.018) in the prepartum period, and by 17% (0.67 ± 0.04 vs. 0.56 ± 0.03 mmol/L, *p* = 0.028) in the postpartum period. Overall, the B-HBA was reduced by 24% (0.77 vs. 0.59 mmol/L, SEM = 0.04, *p* = 0.003) in the whole experimental period (Figure 1; Tables S1 and S2). Mean BW did not differ between the dietary treatments in the whole experimental period (Tables S3 and S4). However, ewes’ BW recovered between lambing and 6th week of lactation tended to increase by 75% (2.00 ± 0.31 vs. 3.44 ± 0.28 kg, *p* = 0.092) in the ActiSaf compared with the Control group (Figure 2; Table S5).

**Figure 3:**
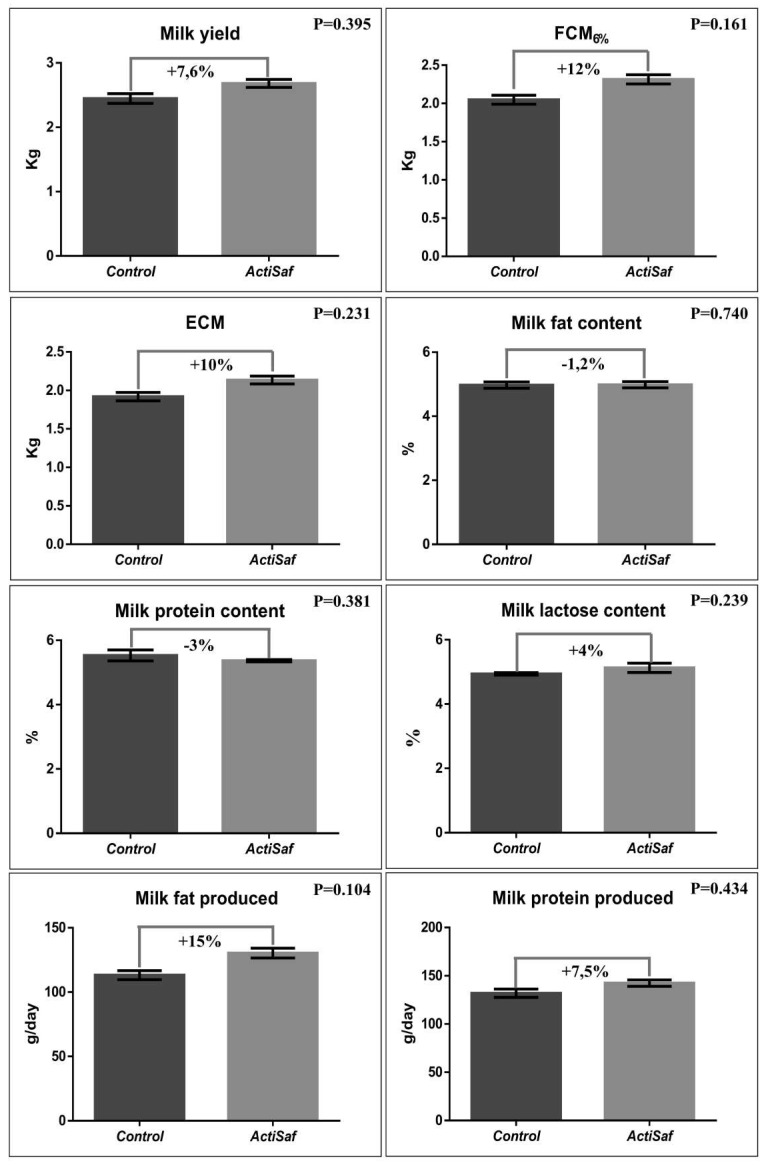
Graphical representation of milk yield and chemical composition of ewes in Control (black, *n* = 51 ewes) and ActiSaf (grey, *n* = 53 ewes) groups (mean ± SEM). FCM: Fat corrected milk in 6% according to the equation Y6% = (0.28 + 0.12F) M, where F = fat% and M = milk yield in kg. ECM: Energy corrected milk = milk yield × (0.071 × fat (%) + 0.043 × protein (%) + 0.2224) [38].

Milk, fat corrected milk 6% (FCM_6%_), and energy corrected milk yield (ECM) were numerically increased by 7.6 (2.50 vs. 2.69 kg/day, SEM = 0.159, *p* = 0.395), 12 (2.07 vs. 2.32 kg/day, SEM = 0.126, *p* = 0.161), and 10% (1.93 vs. 2.13 kg/day, SEM = 0.116, *p* = 0.231), respectively, in the ActiSaf compared to the Control group (Figure 3; Table S6; Figure S1). Concerning milk chemical composition, fat and protein contents were slightly decreased by 1.2 (*p* = 0.740) and 3% (*p* = 0.381), respectively, in the ActiSaf group, due to higher daily milk yield. However, both daily milk fat (114 vs. 131 g/day, SEM = 7.188, *p* = 0.104) and milk protein production (133 vs. 143 g/day, SEM = 8.288, *p* = 0.434) were increased by 15 and 7.5%, respectively, in the ActiSaf group (Figure 3; Supplementation Table S6). The milk yield in the treated ewes showed a moderate increase after the third week in lactation, and a peak in the fourth week, indicating a more intense milk persistence.

### 3.2. Milk Fatty Acid Profile

Milk fatty acid profile was not altered among dietary supplementation except for certain minor differences. Specifically, pentadecanoic acid (C_15:0_), palmitoleic acid (C_16:1_), trans linoleic acid (C_18:2n6t_), and linolenic acid (C_18:3n3_) were increased in ActiSaf milk by 15 (0.82 vs. 0.95%, SEM = 0.045, *p* = 0.042), 13 (0.29 vs. 0.33%, SEM = 0.014, *p* = 0.033), 9 (0.19 vs. 0.22%, SEM = 0.008, *p* = 0.049), and 20% (0.40 vs. 0.48%, SEM = 0.027, *p* = 0.075), respectively, while stearic acid (C_18:0_) decreased by 5% (8.91 vs. 8.44%, SEM = 0.490, *p* = 0.029) (Table 2).

### 3.3. Oxidative Status

**Figure 4:**
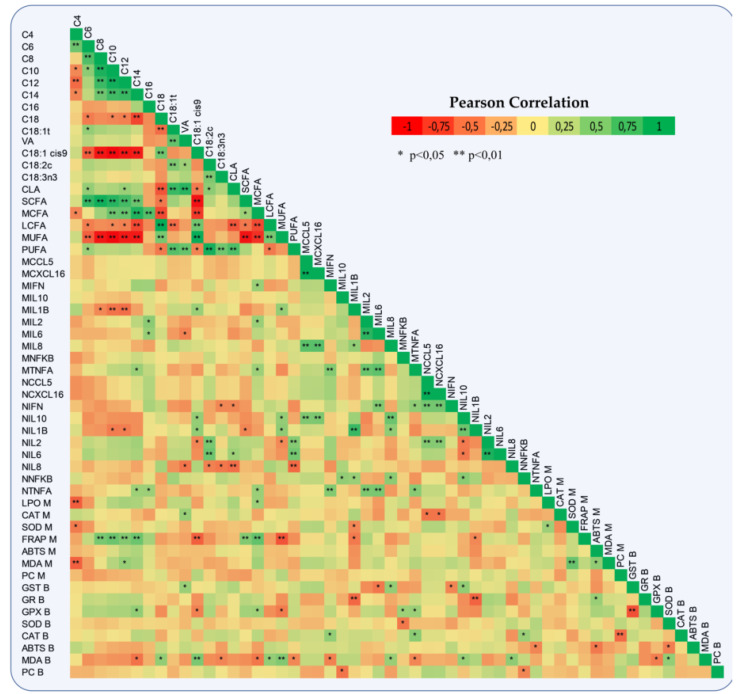
Heat-map represents a Pearson correlation of milk fatty acids, immune system gene expression in monocytes and neutrophils, antioxidant enzymes activities, total antioxidant capacity, and oxidative indices in both blood plasma and milk of ewes. In immune system genes, *M = monocytes and N = neutrophils,* while in antioxidants B = blood plasma and M = milk. CCL5: C-X-C motif chemokine 5, CXCL16: C-X-C motif chemokine ligand 16, INFG: Interferon γ, IL1B: Interleukin-1 beta, IL2: Interleukin-2, IL6: Interleukin-6, IL8: Interleukin-8, IL10: Interleukin-10, TNF: Tumor Necrosis Factor, NFKB: Nuclear Factor kappa B, GST: Glutathione transferase, GR: Glutathione reductase, SOD: Superoxide dismutase, GPx: Glutathione peroxidase, CAT: Catalase, ABTS: 2,2′-Azino-bis (3-ethylbenzthiazoline-6-sulfonic acid), FRAP: Ferric Reducing Ability of Plasma, MDA: Malondialdehyde, PCs: Protein carbonyls, LPO: Lactoperoxidase, VA: Vaccenic acid, CLA: Conjugated linoleic acid, SCFAs: Short-Chain saturated fatty acids, MCFAs: Medium-Chain saturated fatty acids, LCFAs: Long-Chain saturated fatty acids, MUFAs: Mono-unsaturated fatty acids, and PUFAs: Poly-unsaturated fatty acids.

Amongst the dietary treatments, we did not report any significant differences in both blood and milk antioxidant enzymes. However, Glutathione Reductase (GR) in blood plasma was significantly increased by 13% (0.067 vs. 0.076 units/mL, SEM = 0.002, *p* = 0.004) in ActiSaf fed ewes. A numerical increase in lactoperoxidase (LPO) and catalase (CAT) activities by 20 and 10%, respectively, in milk of ActiSaf fed ewes was observed. The total antioxidant capacity measured by ABTS assay was significantly higher by 16.7% (37.563 vs. 43.850% inhibition, SEM = 3.564, *p* = 0.001) in the milk of ActiSaf fed ewes (Table 3). A negative correlation between the total antioxidant capacity determined by FRAP assay and the proportions of MUFA and oleic acid (C*_18:1 cis-9_*) in milk was found. The same trend was reported between Glutathione Peroxidase (GPx) activity in blood plasma and the aforementioned fatty acids of milk. The correlation between blood malondialdehyde (MDA) content and the proportions of milk’s PUFA was also negative. On the other hand, the correlations between blood MDA content and the proportions of MUFA and oleic acid, respectively, were positive (Figure 4).

### 3.4. Immune Status

The relative transcript levels of both *CCL5* and *CXCL16* in monocytes of ActiSaf fed ewes were significantly suppressed by 30% (0.053 vs. 0.037, SEM = 0.003, *p* = 0.007 and 0.042 vs. 0.029, SEM = 0.008, *p* = 0.008, respectively) (Table 4). Amongst cytokines, Interleukin 8 (*IL8*) relative transcript levels were significantly decreased by 80% (0.0020 vs. 0.0004, SEM = 0.0000, *p* = 0.031), while Interleukin 1β (*IL1B*) showed a tendency to decrease by 43% (0.007 vs. 0.004, SEM = 0.001, *p* = 0.087) in ActiSaf fed ewes (Table 4). A significant downregulation in the relative expression of Interleukin 10 (*IL10*) by 30% (0.014 vs. 0.010, SEM = 0.002, *p* = 0.047) in the neutrophils of the treated ewes was observed (Table 4). In addition, the relative transcript levels of *IL1B* in monocytes were positively correlated with the proportions of both MUFA and oleic acid in milk. The same trend was found between the relative expression of the Interleukin 2 gene and the proportion of palmitic acid in milk (Figure 4). Monocytes and neutrophil relative transcript levels of *IL1B* were negatively correlated with GR and Glutathione Transferase (GST) activities in blood plasma, respectively (Figure 4).

### 3.5. Holistic Statistics

**Figure 5:**
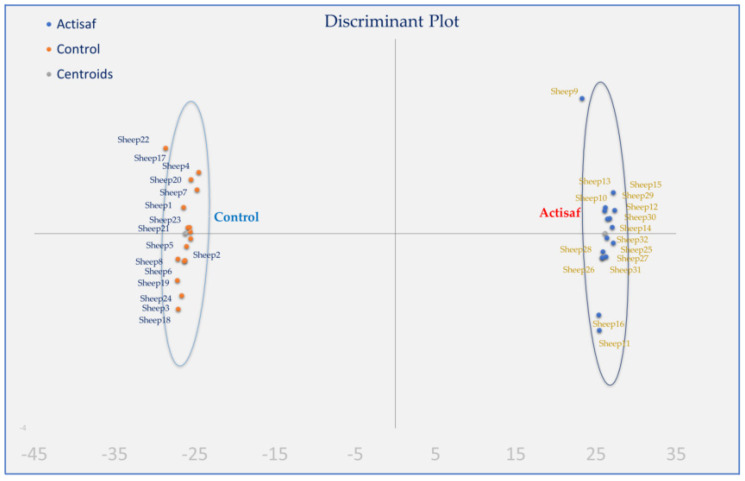
Discriminant plots separating the Control and ActiSaf fed ewes according to their fatty acid grouped values, immune gene expression in both monocytes and neutrophils, and antioxidant indices in blood and milk.

Discriminant analysis was applied to pooled data of two sampling times (3rd and 6th week postpartum) according to fatty acids in milk, immune gene expression in both monocytes and neutrophils, and antioxidant indices in blood plasma and milk (Figure 5) to investigate if the samples can be distinguished according to the type of the diet (Control and ActiSaf). The percentages of the samples that were classified into the correct group, according to the dietary treatment, were 100%. Wilks’ lambda was observed at 0.001 for Function 1 (*p* = 0.159), while the relative transcript levels of *CCL5, CXCL16, and IL6* in monocytes’, *IL10, IL6,* and *IL8* in neutrophils, and the GR activity in blood plasma were the variables that contributed the most.

## 4. Discussion

Blood B-HBA concentrations reflect the magnitude of negative energy balance (NEB) and lipid mobilization and are a diagnostic marker for subclinical (SCK) and clinical ketosis (CK) in ruminants. The B-HBA content in the blood of sheep with SCK ranges from 0.5 to 1.6 mmol/L [39,40,41,42], while in those with CK from 1.6 to 7 mmol/L [39,40,43]. However, in the case of healthy pregnant sheep these values could be around 0.8–0.9 mmol/L [44,45]. Nonetheless, the values of the Control group, first and foremost during the prepartum period, may indicate a moderate NEB (0.86 mmol/L). On the other hand, results regarding the B-HBA concentration in the blood of ActiSaf fed ewes indicate an improvement in the energetic status of the animals. It should be underlined here that the prolificacy between the two groups was the same (around 2.35), which means that although the number of fetuses affects B-HBA content [46], it had minimum impact in our trial. The same levels of B-HBA content in blood of healthy ewes of the same breed in early lactation have been previously reported [18,38]. An increase in the host energy availability might be due to a better rumen function and microbiome homeostasis. During the peripartum period, the energy and nutrient demands increase exponentially while the dry matter intake decreases. Thus, the optimal rumen function and the balance between VFA for a maximum feed efficiency are momentous in the transition period. The mechanism underling LY contribution in rumen may be down to yeast’s oxygen scavenging properties (anaerobiosis). Specifically, the improvement of anaerobiosis in rumen increases the bacteria viability and thus, microbial protein synthesis and fiber digestibility [6]. Energy balance might be improved as a result of the dry matter (DM) and NDF digestion enhancement as have been reported by Plata et al. [47]. Furthermore, Panda et al. [48] also found that crude protein (CP) digestibility was also increased by 4.8% with dietary supplementation of yeast culture on male calves. In agreement with our findings, ActiSaf dietary inclusion (5g/day) in early lactating dairy cows, significantly decreased serum B-HBA and non-esterified fatty acids (NEFA) concentrations [9], while in mid-lactating cows, 4 g/day of LY supplementation did not affect B-HBA concentration since animals were not prone to NEB [49]. In addition, ewes in the ActiSaf group recovered their body weight from lambing until the sixth week postpartum in a more efficient manner, considering the increased available energy, as concluded by B-HBA concentration.

In compliance with our findings (12% FCM_6%_, *p* = 0.161), Stella et al. [12], reported a significant increase in goats’ milk yield by 14% when their diet was supplemented with *S. cerevisiae*. It is worth mentioning that, in the same study, treated goats showed an upward trend in milk yield after the fourth week postpartum which decreased slower compared to the control group, showing a persistence in milk similar to our study. The dietary inclusion of *S. cerevisiae* enhanced cows’ milk yield in early [13] mid- [14] and late [7] lactation. However, Dehghan-Banadaky et al. [49], showed that the milk yield was not affected in *S. cerevisiae* supplemented cows after the 145 DIM, possibly due to the absence of NEB. The results from 22 studies with more than 9039 lactating dairy animals showed an increase in their milk production by 7.3% (ranging from 2 to 30%) when their diets were supplemented with Yea-Sacc^®^1026 yeast [50].

Interestingly, in a meta-analysis study, Dehghan-Banadaky [49] concluded that an enhancement in milk yield was accompanied by an increase in feed intake in supplemented animals with yeast products. Moreover, yeast administration in prepartum cows’ diets improved DMI [51]. Additionally, Habeeb et al. [52] reported that an enhancement of animal performance by the inclusion of yeast in their diet was mainly attributed to an increase in feed intake rather than feed digestibility improvement.

The milk fatty acid’s profile was not holistically modified; however, certain interesting results, related to the biohydrogenation process (BH), were unveiled. Julien et al. [53] first observed the impact of LY administration on ruminal biohydrogenation processes. Specifically, LY promotes growth and activity of rumen lactate-utilizing bacteria, such as *Megasphera elsdenii* or *Selenomonas ruminantium*, *Actinobacteria*, including *Propionibacterium acnes* as well as fibrolytic bacteria. Consequently, LY could be involved at different stages of BH; firstly, by altering biohydrogenating microorganisms, i.e., improving growth of either t11 or t10 isomer producing bacteria, and secondly by modulating the ruminal biotope, i.e., by stabilizing ruminal pH or favoring stronger reducing conditions. In addition, Julien et al. [53] reported that LY supplementation increased the accumulation of trans C_18:1_ in vitro and decreased the proportion of C_18:0_, suggesting an inhibition of the last step of BH of _c9c12_-C_18:2_ fatty acids. Thus, in our study it could be hypothesized that the improved rumen conditions by LY administration may favor the isomerisation of _c9c12-_C_18:2_ and consequently increased the production of intermediate fatty acids in the rumen, which induced an inhibition or a saturation of the enzyme activity of bacteria involved in the second reduction step [54].

During the peripartum period, animals’ augmented requirement for energy and nutrient results in lipid mobilization and blood hyperketonemia which induce oxidative stress [55]. Optimizing nutrition requirements by improving rumen efficiency may suppress the concentration of such trigger metabolites and improve the oxidative status. Glutathione reductase (GR) has a central role in the antioxidant defense system since it catalyzes the conversion of oxidized glutathione disulfide to the reduced form of glutathione, which is a critical molecule in resting oxidative stress [56]. It is known that glutathione is extremely important since it acts as substrate or co-substrate in enzymatic reactions (e.g., the glutathione-S-transferase or glutathione-shuttle enzymes), reacts directly with free radicals and lipid peroxides, and protects cells [57]. The mechanism under which GR increased its activity remains unclear, thus our assumptions are oriented toward a prudent liver function where glutathione is de novo synthesized due to the lower B-HBA concentration. Another possible mechanism that can increase GR activity might be the Flavin Adenine Dinucleotides’ (FADs) co-substrate. Specifically, yeasts are sources of B-complex vitamins that act as precursors of the essential co-enzymes NAD and FAD that are responsible for biological oxidation [58]. In addition, high genetic merit dairy animals often burden their metabolism since they require increased levels of energy in order to meet their demands, leading to ROS production and later to the annihilation of the milk oxidative stability [59]. Hence, total antioxidant capacity enhancement in the milk of ActiSaf fed ewes may be important for the dairy industry. 

Concerning chemokines, CCL-5 is involved in the activation of T cells, macrophages, eosinophils and basophils, and its enhancement is related to an inflammation response [60]. On the other hand, CXCL-16, a transmembrane protein, is detached from the membrane by metalloproteinase ADAM10 induced chemotaxis [61]. In this study, the downregulation in the relative transcript levels of both *CCL5* and *CXCL16* in monocytes of ActiSaf treated ewes, indicates a lower inflammatory response during the first 6 weeks of lactation. The *IL1B* which was suppressed in our study, regulates B-cell maturation and proliferation, activates the Natural Killer (NK) cells and is generally related to the acute manifestation of inflammation in immune cells [62]. Interleukin-8, on the other hand, has a chemotaxis-inducer effect mainly in neutrophils. Pro-inflammatory chemokines and cytokine downregulations is directly attributed to B-HBA mitigation. Specifically, blood ketones derived from ketogenesis through acetyl-CoA metabolization have been shown to act as stimulants in chemokines and cytokines in cows’ mammary epithelia cells [63].

## 5. Conclusions

In conclusion, supplementing dairy sheep diets with 2 g of the ActiSaf live yeast/day/ewe during the transition and early lactation periods have a beneficial impact on animals’ performance whilst simultaneously portraying an improvement on pro-inflammatory responses attributed to a lower lipomobilization. This overall stress suppression during this turning point for the ruminants’ period may unveil the potential of live yeasts as health modulators towards the collective effort of reducing antibiotic dependance at the farm scale. However, further research is needed to deeply understand the mechanism under the enhancement of energy supply in small ruminants.

## Figures and Tables

**Table 1:** Concentrates composition (g/kg), diet intake (g), daily nutrients intake (g/ewe), and feeds chemical composition and fatty acid profile (%).

Ingredients (g/kg)	Concentrates			
	Control		ActiSaf	
Maize grain	575		575	
Wheat middlings	180		178	
Soybean meal	220		220	
Mineral and vitamin	25		25	
ActiSaf	-		2	
**Daily feed intake (g/day/ewe)**				
	Prepartum	Postpartum	Prepartum	Postpartum
Oat hay	700	700	700	700
Alfalfa hay	700	700	700	700
Con. Mix	1000	2000	-	1000
ActiSaf Mix	-	-	1000	1000
**Nutrients intake (g/day/ewe)**				
Dry matter	2093	2956	2091	2953
Crude protein	318	480	318	481
Ether extract	46	69	44	66
NDF	835	991	835	992
ADF	562	626	561	624
**Diet chemical composition (%)**				
	Con. Mix	ActiSaf Mix	Alfalfa hay	Oat hay
Dry matter	86.28	86.02	88.21	87.63
Organic matter	81.70	81.16	80.87	80.91
Crude protein	16.23	16.29	14.72	7.38
Ether extract	2.22	2.01	1.38	2.06
NDF	15.62	15.68	43.25	53.68
ADF	6.32	6.19	32.56	38.74
Ash	4.58	4.86	73.4	6.72
**Fatty acids composition (%)**				
C_14:0_	0.1	0.1	0.6	5.6
C_16:0_	14.3	14.3	23.9	3.7
C_18:0_	3.9	3.9	3.1	19.5
*cis_-9_* C_18:1_	36.1	36.1	3.1	16.2
C_18:2 n-6_	42.2	42.2	21.3	54.0
C_18:3 n-3_	1.7	1.7	41.9	1.2

NDF = Neutral detergent fiber; ADF = Acid detergent fiber.

**Table 2:** The mean individual fatty acids (FAs) (% of total FA), FA groups and Saturated Fatty Acids (SFAs)/Unsaturated Fatty Acids (UFAs) of milk from ewes fed Control (*n* = 8 ewes) and ActiSaf (*n* = 8 ewes) diet throughout the experimental period (21 and 42 experimental days).

	Diets (D)			Sampling Time (T) in Weeks			Effect *		
	Control	ActiSaf	SEM ^†^	3	6	SEM ^†^	D	T	DxT
C_4:0_	4.81	4.59	0.288	4.79	4.61	0.205	0.522	0.230	0.989
C_6:0_	3.75	3.60	0.191	3.42	3.93	0.181	0.788	0.005	0.589
C_8:0_	3.17	3.12	0.171	2.90	3.89	0.125	0.922	0.010	0.489
C_10:0_	9.08	9.14	0.639	8.05	10.17	0.451	0.789	0.001	0.367
C_12:0_	4.88	5.01	0.395	4.33	5.56	0.285	0.422	0.000	0.148
C_14:0_	10.84	11.16	0.325	10.41	11.58	0.251	0.398	0.000	0.257
C_14:1_	0.24	0.30	0.030	0.26	0.28	0.028	0.152	0.890	0.789
C_15:0_	0.82	0.95	0.045	0.83	0.94	0.037	0.042	0.004	0.174
C_15:1_	0.24	0.29	0.032	0.28	0.25	0.027	0.259	0.520	0.258
C_16:0_	23.94	24.90	0.596	24.10	24.42	0.428	0.189	0.890	0.478
C_16:1_	0.29	0.33	0.014	0.309	0.314	0.011	0.033	0.621	0.585
C_17:0_	0.60	0.65	0.031	0.701	0.544	0.020	0.698	0.003	0.984
C_17:1_	0.31	0.34	0.030	0.40	0.25	0.022	0.980	0.000	0.970
C_18:0_	8.91	8.44	0.490	8.86	8.48	0.352	0.029	0.045	0.368
Σ*_trans_* C_18:1_	1.07	1.01	0.110	1.05	1.02	0.088	0.893	0.880	0.489
^‡^*_trans-11_* C_18:1_	1.26	1.16	0.158	1.22	1.20	0.125	0.358	0.499	0.984
*cis*_-9_ C_18:1_	20.99	20.05	1.260	23.09	17.97	0.785	0.639	0.002	0.874
C_18:2n6t_	0.19	0.22	0.008	0.19	0.22	0.008	0.049	0.019	0.321
C_18:2n6c_	3.02	3.09	0.213	2.84	3.26	0.168	0.890	0.009	0.284
C_20:0_	0.12	0.12	0.004	0.11	0.13	0.006	0.980	0.033	0.678
C_18:3n3_	0.40	0.48	0.027	0.45	0.43	0.020	0.075	0.459	0.574
C_20:3n3_ + C_22:1_	0.31	0.28	0.015	0.34	0.25	0.012	0.784	0.000	0.348
_trans-11_, _cis-9_ C_18:2_	0.76	0.77	0.110	0.74	0.80	0.081	0.899	0.269	0.635
^§^SCFA	20.81	20.45	0.896	19.16	22.10	0.678	0.678	0.002	0.354
^¶^ΜCFA	41.07	42.07	0.858	40.69	43.04	0.689	0.201	0.008	0.528
^††^LCFA	9.03	8.56	0.489	8.97	8.62	0.361	0.302	0.099	0.598
^‡‡^ΜUFA	24.40	23.48	1.140	26.61	21.28	0.651	0.522	0.008	0.789
^§§^ PUFA	4.68	4.84	0.308	4.57	4.95	0.212	0.622	0.028	0.654
^¶¶^ SFA	70.91	71.67	1.046	68.82	73.75	0.721	0.589	0.007	0.354
^†††^UFA	29.09	28.33	1.046	31.18	26.25	0.712	0.453	0.007	0.352
^‡‡‡^SFA/UFA	2.43	2.53	0.143	2.21	2.80	0.086	0.870	0.006	0.123
^§§§^AI	2.56	2.68	0.133	2.33	2.92	0.099	0.256	0.002	0.099

* Effect: The dietary treatment (D), time (T), and the interaction between dietary treatment × time (DxT) effects were analyzed by analysis of variance (ANOVA) using a general linear model (GLM) for repeated measures and posthoc analysis was performed when appropriate using Tukey’s multiple range test. ^†^SEM = Standard error of the mean. ^‡^*_trans-11_* C_18:1_ = these values are not included in the Σ *_trans_* C_18:1_ content. ^§^SCFAs: Short-Chain Saturated Fatty Acids = C_6:0_ + C_8:0_ + C_10:0_ + C_11:0_; ^¶^MCFAs: Medium-Chain Saturated Fatty Acids = C_12:0_ + C_13:0_ + C_14:0_ + C_15:0_ + C_16:0_ + C_17:0._^††^LCFAs: Long-Chain Saturated Fatty Acids = C_18:0_ + C_20:0_. ^‡‡^MUFAs: Mono-Unsaturated Fatty Acids = C_14:1_ + C_15:1_ + C_16:1_ + C_17:1_ + C_18:1 *cis-9*_ + *_trans-11_* C_18:1_ + *_trans_* C_18:1_; ^§§^PUFAs: Poly-Unsaturated Fatty Acids = *_cis-9, trans-11_* C_18:2_ (CLA) + C_18:2n-6c_ + C_18:2n-6t_ + C_18:3n-3_ + C_18:3n-6_ + C_20:3n-3_; ^¶¶^SFAs: Saturated Fatty Acids = SCFA + MCFA + LCFA; ^†††^UFAs: Unsaturated Fatty Acids = PUFA + MUFA; ^‡‡‡^S/U: Saturated/Unsaturated = (SCFA + MCFA + LCFA)/(PUFA + MUFA), and ^§§§^AI: Atherogenicity index = (C_12:0_ + 4 * C_14:0_ + C_16:0_)/(PUFA + MUFA).

**Table 3:** Enzymes activities (Units/mL), total antioxidant capacity, and oxidative status biomarkers in blood plasma and milk of ewes fed the two diets (Control, *n* = 8 and ActiSaf, *n* = 8) at two sampling times.

	Diets (D)			Sampling Time (T) in Weeks			Effect *		
	Control	ActiSaf	SEM ^†^	3	6	SEM ^†^	D	T	DxT
Blood plasma									
GST	0.150	0.132	0.011	0.148	0.134	0.011	0.282	0.336	0.903
GR	0.067	0.076	0.002	0.067	0.076	0.002	0.004	0.003	0.458
GSH-Px	0.398	0.423	0.025	0.353	0.468	0.021	0.476	0.000	0.847
SOD	18.544	20.304	0.987	18.409	20.440	1.170	0.227	0.301	0.427
CAT	4.969	5.008	0.146	4.893	5.084	0.131	0.855	0.255	0.477
FRAP	0.641	0.764	0.045	0.675	0.730	0.036	0.099	0.230	0.409
ABTS	36.888	33.045	0.497	35.577	34.356	0.617	0.000	0.246	0.195
MDA	0.601	0.566	0.067	0.604	0.562	0.055	0.729	0.497	0.304
PC	4.065	4.015	0.085	3.886	4.193	0.110	0.685	0.114	0.981
Milk									
LPO	0.504	0.602	0.059	0.628	0.478	0.047	0.265	0.006	0.467
SOD	3.677	3.569	0.173	3.577	3.669	0.157	0.664	0.660	0.859
CAT	29.284	32.359	3.564	33.558	28.085	2.836	0.552	0.054	0.750
FRAP	3.013	3.090	0.179	2.855	3.247	0.158	0.766	0.078	0.445
ABTS	37.563	43.85	1.014	40.254	41.158	1.057	0.001	0.572	0.726
MDA	0.363	0.369	0.018	0.363	0.369	0.015	0.843	0.693	0.687
PC	2.916	2.877	0.106	2.925	2.867	0.108	0.801	0.718	0.541

^†^ SEM = Standard error of the mean. GST: Glutathione transferase. GR: Glutathione reductase. SOD: Superoxide dismutase. GSH-Px: Glutathione peroxidase. CAT: Catalase. ABTS: 2,2′-Azino-bis (3-ethylbenzthiazoline-6-sulfonic acid) (inhibition%). FRAP: Ferric Reducing Ability of Plasma (μΜ ascorbic acid). MDA: Malondialdehyde (μΜ MDA). PCs: Protein carbonyls (nmol/mL). LPO: Lactoperoxidase. Significance level below 0.05 indicates significant difference. * Effect: The dietary treatment (D), time (T), and the interaction between dietary treatment × time (DxT) effects were analyzed by ANOVA using a general linear model (GLM) for repeated measures and posthoc analysis was performed when appropriate using Tukey’s multiple range test.

**Table 4:** Relative transcript levels of several genes in blood monocytes and neutrophils of ewes fed the two diets (Control, *n* = 8 and ActiSaf, *n* = 8) at two sampling times.

	Diets (D)			Sampling Time (T) in Weeks			Effect *		
	Control	ActiSaf	SEM ^†^	3	6	SEM ^†^	D	T	DxT
Monocytes									
*CCL5*	0.053	0.037	0.003	0.047	0.043	0.004	0.007	0.621	0.030
*CXCL16*	0.042	0.029	0.008	0.037	0.034	0.004	0.008	0.599	0.032
*IFNG*	0.009	0.008	0.001	0.006	0.010	0.001	0.301	0.034	0.856
*IL1B*	0.007	0.004	0.001	0.007	0.004	0.001	0.087	0.010	0.016
*IL2*	0.005	0.006	0.001	0.004	0.007	0.001	0.484	0.147	0.837
*IL6*	0.002	0.002	0.000	0.001	0.002	0.000	0.896	0.061	0.786
*IL8*	0.0020	0.0004	0.0000	0.0020	0.0004	0.0000	0.031	0.007	0.007
*IL10*	0.019	0.018	0.002	0.020	0.017	0.002	0.727	0.126	0.383
*TNF*	0.053	0.048	0.003	0.040	0.061	0.003	0.266	0.000	0.886
*NFKB*	0.251	0.245	0.022	0.254	0.242	0.024	0.842	0.727	0.708
Neutrophils									
*CCL5*	0.083	0.079	0.014	0.067	0.095	0.014	0.854	0.179	0.356
*CXCL16*	0.069	0.065	0.012	0.055	0.079	0.012	0.856	0.190	0.355
*IFNG*	0.026	0.015	0.007	0.018	0.023	0.005	0.289	0.424	0.719
*IL1B*	0.014	0.010	0.002	0.017	0.008	0.002	0.171	0.003	0.022
*IL2*	0.023	0.021	0.004	0.011	0.033	0.003	0.703	0.003	0.975
*IL6*	0.007	0.006	0.002	0.001	0.012	0.002	0.821	0.005	0.642
*IL8*	0.003	0.004	0.001	0.004	0.003	0.001	0.781	0.142	0.119
*IL10*	0.014	0.010	0.002	0.016	0.008	0.002	0.047	0.007	0.032
*TNF*	0.298	0.442	0.052	0.262	0.478	0.062	0.075	0.061	0.578
*NFKB*	0.182	0.185	0.028	0.239	0.0128	0.025	0.935	0.018	0.359

Significance level below 0.05 indicates significant difference. ^†^ SEM = Standard error of the mean. *CCL5*: C-X-C motif chemokine 5. *CXCL16*: C-X-C motif chemokine ligand 16. *INFG*: Interferon γ. *IL1B*: Interleukin-1 beta. *IL2*: Interleukin-2. *IL6:* Interleukin-6. *IL8*: Interleukin-8. *IL10*: Interleukin-10. *TNF*: Tumor Necrosis Factor. *NFKB*: Nuclear Factor kappa B. * Effect: The dietary treatment (D), time (T), and the interaction between dietary treatment × time (DxT) effects were analyzed by ANOVA using a general linear model (GLM) for repeated measures and posthoc analysis was performed when appropriate using Tukey’s multiple range test.

## Supplementary Materials

The following are available online at https://www.mdpi.com/2309-608X/6/4/334/s1, Table S1. Analysis of variance in blood β-hydroxybutyric acid in prepartum and postpartum period; Table S2. Repeated measure analysis of variance in blood β-hydroxybutyric acid in overall experimental period using prepartum and postpartum sampling time as repeated factor; Table S3. Analysis of variance in body weight at the start of the experiment, at lambing, and at the end; Table S4. Repeated measure analysis of variance in body weight in overall experimental period using the weighing at the start of the experiment, at lambing and at the end, as repeated factor.; Table S5. Analysis of variance in body weight recovery from lambing to 6 weeks postpartum. Table S6. Milk yield and milk chemical composition of ewes fed the Control and ActiSaf diets in the six sampling times; Table S7. Sequences of primers for target genes used in real-time qPCR; Figure S1. Graphical representation of milk chemical composition of the Control and ActiSaf groups.
