# Supplementary material for: Dietary Supplementation of a Live Yeast Product on Dairy Sheep Milk Performance, Oxidative and Immune Status in Peripartum Period
Source: J Fungi (Basel). 2020 Dec 3;6(4):334. doi: 10.3390/jof6040334 (PMC7761757; doi:10.3390/jof6040334)
Supplement: Supplementary file 1 [file jof-06-00334-s001.pdf]

Table S1. Analysis of variance in blood β-hydroxybutyric acid in prepartum and postpartum period.

	Control		ActiSaf		Effect
	$\bar{X}$	± Std Error	$\bar{X}$	± Std Error	Sig.
Prepartum day	-15.3	0.55	-17.0	0.49	0.220
B-BHA mmol/L	0.86[a]	0.07	0.63[b]	0.06	**0.018**
Postpartum day	+11.5	0.33	+11.9	0.52	0.520
B-BHA mmol/L	0.67[a]	0.04	0.56[b]	0.03	**0.028**

Significance level below 0.05 indicates significant difference between treatments.

Table S2. Repeated measure analysis of variance in blood β-hydroxybutyric acid in overall experimental period using prepartum and postpartum sampling time as repeated factor.

	Diets (D)			Sampling Time (T)			Effect		
	Control	ActiSaf	SEM	Prepartum	Postpartum	SEM	D	T	DxT
B-BHA mmol/L	0.77[a]	0.59[b]	0.041	0.74[a]	0.61[b]	0.033	**0.003**	**0.016**	0.263

Significance level below 0.05 indicates significant difference between treatments.

Table S3. Analysis of variance in body weight at the start of the experiment, at lambing, and at the end.

	Control		ActiSaf		Effect
	$\bar{X}$	± Std Error	$\bar{X}$	± Std Error	Sig.
BW start (Kg)	61.5	1.42	61.5	1.41	0.997
BW lambing (Kg)	58.5	0.97	57.6	1.17	0.566
BW end (Kg)	60.5	0.97	61.0	1.11	0.697

Significance level below 0.05 indicates significant difference between treatments, while those close to 0.999 no significant.

Table S4. Repeated measure analysis of variance in body weight in overall experimental period using the weighing at the start of the experiment, at lambing, and at the end, as repeated factor.

	Diets (D)			Sampling Time (T)				Effect		
	Control	ActiSaf	SEM	Start	Lambing	End	SEM	D	T	DxT
BW (Kg)	60.1	60.0	1.09	61.5[b]	58.0[a]	60.8[b]	0.73	0.950	**0.000**	0.442

Significance level below 0.05 indicates significant difference between treatments, while those close to 0.999 no significant.

Table S5. Analysis of variance in body weight recovery from lambing to 6 weeks postpartum.

	Control		ActiSaf		Effect
	$\bar{X}$	± Std Error	$\bar{X}$	± Std Error	Sig.
BW recovery (Kg)	2.00	0.31	3.44	0.28	0.092

Significance level below 0.05 indicates significant difference between treatments, while those close to 0.999 no significant.

Figure S1. Graphical representation of milk chemical composition of the Control and ActiSaf group. Error bars represent the Standard Error of Means.

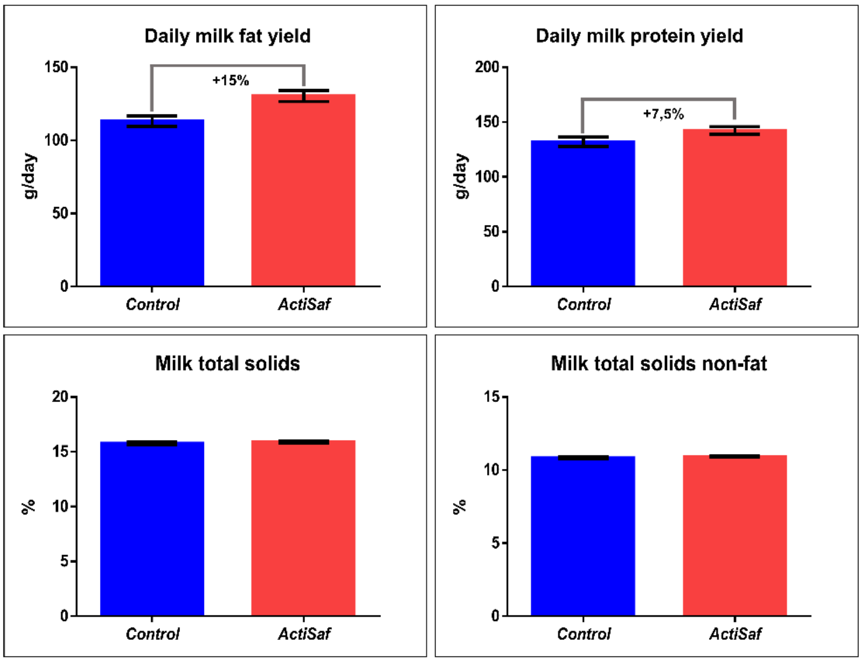

Table S6. Milk yield and milk chemical composition of ewes fed the Control diet and ActiSaf in the six sampling times.

	Diets (D)			Sampling Time (T) in weeks							Effect		
	Control	ActiSaf	SEM	1	2	3	4	5	6	SEM	D	T	DxT
Milk yield Kg	2.50	2.69	0.159	2.02^{a}	2.46^{b}	2.67^{c}	2.90^{d}	2.79^{e}	2.71^{c}	0.127	0.395	0.000	0.001
$FCM_{6\%}$ Kg	2.07	2.32	0.126	2.20^{ad}	2.00^{b}	2.16^{ac}	2.34^{d}	2.21^{cd}	2.24^{cd}	0.103	0.161	0.003	0.131
ECM Kg	1.93	2.13	0.116	1.94^{ab}	1.88^{a}	2.02^{b}	2.21^{c}	2.08^{bd}	2.00^{d}	0.093	0.231	0.000	0.141
Fat %	4.94	5.00	0.117	6.90^{a}	4.67^{b}	4.54^{b}	4.55^{b}	4.43^{b}	4.71^{b}	0.138	0.740	0.000	0.239
Fat g/day	114	131	7.188	136^{ad}	110^{b}	117^{bc}	128^{cd}	119^{cd}	123^{dc}	6.240	**0.104**	0.000	0.056
Protein %	5.54	5.37	0.131	6.43^{a}	5.36^{b}	5.17^{c}	5.27^{cd}	5.16^{c}	5.32^{bd}	0.045	0.381	0.000	0.100
Protein g/day	133	143	8.228	123^{ab}	130^{a}	137^{b}	152^{c}	142^{c}	143^{c}	6.599	0.434	0.000	0.355
Lactose %	4.94	5.13	0.104	4.61^{a}	5.39^{abc}	4.99^{b}	5.03^{b}	5.07^{c}	5.12^{bc}	0.083	0.239	0.000	0.284
TS %	15.73	15.89	0.151	18.03^{a}	15.51^{b}	15.23^{b}	15.38^{b}	15.13^{b}	15.59^{b}	0.159	0.470	0.000	0.346
TSNF %	10.81	10.91	0.058	10.98	10.85	10.73	10.84	10.79	10.94	0.044	0.261	0.116	0.762

Table S7. Sequences of primers for target genes used in real-time qPCR.

Gene	Sequence	Accession No.*
GAPDH	F: 5'-AAAGCCATCACCATCTTCCA -3'<br>R: 5'-ACCACGTACTCAGCACCTCAT-3'	NM_001190390.1
YWHAZ	F: 5'-TGTTCTATTGTGCCTAGTACACTGT -3'<br>R: 5'-CATCAAGACTCACTGCCTCCC -3'	NM_001267887.1
IL-1β	F: 5'-TGGATAGCCCATGTGTGCTG -3'<br>R: 5'-CAGAACACCACTTCTCGGCT -3'	NM_001009465.2
IL2	F: 5'-AAATCCCGAGAACCTCAAGCT -3'<br>R: 5'-TGTAGCGTTAACCTTGGGCA -3'	NM_001009806.1
IL6	F: 5'-CAGCAAGGAGACACTGGCAGA-3'<br>R: 5'-TCCATCTTTTTCCTCCATTTTTGG -3'	NM_001009392.1
IL8	F: 5'-CCTGCTCTCTGCAGCTCTGTG-3'<br>R: 5'-TGCATTGGCATCGAAGTTCTG -3'	NM_001009401.2
IL10	F: 5'-CTGGGGGAGAAGCTGAAGAC-3'<br>R:5'-CTCTCTTCACCTGCTCCACC-3'	NM_001009327.1
IFNg	F: 5'-AAATTCCGGTGGATGATCTG -3'<br>R: 5'-ACCATTACATTGATGCTCTCC -3'	NM_001009803
TNFa	F: 5'-GGGAGACACAAACTAAGGGCT -3'<br>R: 5'-AACCTGCAGTTCAGCTCCG -3'	NM_001024860
NF-kB	F: 5'-AAGCTGTGGTGGAGGACTTG -3'<br>R: 5'-ACAGAGTTACCCAAGCGGTC -3'	AF283892.1
CXCL-5	F: 5'-CAAGTGCTCCATGGCAGCAG -3'<br>R: 5'-GTTGGCGCACACCTGACG -3'	XM_004012452.3
CXCL-16	F: 5'-GTGCCTGTGTTGTCCCTCTT -3'<br>R: 5'-GCTTGCACACCACGTAGAGT -3'	XM_015098600.1

*Ref Seq: NCBI Reference Sequence database
